# Methylation of LINE-1 in cell-free DNA serves as a liquid biopsy biomarker for human breast cancers and dog mammary tumors

**DOI:** 10.1038/s41598-018-36470-5

**Published:** 2019-01-17

**Authors:** Kang-Hoon Lee, Tae-Jin Shin, Wan-Hee Kim, Je-Yoel Cho

**Affiliations:** 1 Department of Biochemistry, BK21 Plus and Research Institute for Veterinary Science, School of Veterinary Medicine, Seoul National University, Seoul, South Korea; 2 Department of Veterinary Clinical Sciences, College of Veterinary Medicine and Research Institute for Veterinary Science, Seoul National University, Seoul, Republic of Korea

**Keywords:** Breast cancer, Diagnostic markers

## Abstract

Breast cancer (BC) is one of the most common cancers in both women and female dogs. Methylation changes of LINE-1 have been reported in human cancers. The aim of this study was to determine the hypomethylation of canine LINE-1 in liquid biopsies for canine mammary tumors (CMT) and to assess its diagnostic performance in human plasma. BC associated LINE-1 methylation was measured by methylation sensitive (HpaII) and insensitive (MspI) restriction enzyme digestion followed by real-time PCR using the cfDNA isolated from 300 µl of plasma. The relative level of methylated canine LINE-1 was less than 0.4 in the benign and malignant CMTs (0.29 ± 0.061 and 0.39 ± 0.066, respectively) when it was 0.92 ± 0.067 in the healthy controls. The area under the ROC curve (AUC) was significantly high in both benign and malignant tumors (0.97 and 0.93). Furthermore, this approach was also successfully implemented in a set of 26 human BCs with 10 healthy controls (AUC = 0.78). Altogether, our data suggest that the comparative approach using a dog model might be helpful to rapidly develop a new diagnostic biomarker and that the methylation of LINE-1 in cfDNA may be a good target as a diagnostic marker of both human BC and CMT.

## Introduction

In women, human breast cancer (HBC) is the most frequently diagnosed cancer and accounts for the highest cancer mortality^1^. Although several molecular biomarkers have been identified, medical imaging methods such as mammography are still the standard method for the early detection of HBC in the clinic^2^. This is because imaging methods are effective for detecting HBC in reasonably early stages. However, it has limitations in accuracy depending on the density of the breast tissues, resulting in less sensitivity among younger and Asian women, and depending on the location of the cancer^3^. Moreover, HBC screening using mammograms requires additional tests to look at suspicious regions more closely. Thus, the identification and validation of diagnostic biomarkers for HBC and HBC subtypes from liquid biopsy in the clinic are still necessary.

Due to the similarity between HBC and canine mammary tumors (CMTs), including the high incidence and malignancy of CMT, CMT is well known as a good animal model for HBC. Additionally, the importance of understanding CMT is higher than ever. Since 1970, when Robert Schneider published a paper entitled “Comparison of age, sex, and incidence rates in human and canine breast cancer”, many studies have documented that there is a great deal of similarity between the two regarding molecular marker expression, hormone dependency and cancer phenotypes^4^. The counterparts of HBC molecular markers and certain cell types of HBCs, such as myoepithelial cells which are rare in human breast cancer, have been investigated genetically and epigenetically in the canine model^5,6^. Moreover, the partially fixed backgrounds of dogs serve as an advantage in the use of CMT as a model for HBC, considering the complexity in a human study with all of its disorders and environmental effects. Therefore, development of diagnostic biomarkers using CMTs might be a very effective approach for studying HBC and HBC subtypes.

Circulating cell-free DNA (cfDNA) has been validated as a blood-based biomarker of various cancers in a number of studies^7,8^. Various tumor-associated aberrations, including single nucleotide polymorphisms, copy number variations^9^, cfDNA integrity^10^, and epigenetic modifications such as DNA methylation and histone modification have been reported^11,12^. All analysis platforms have both advantages and limitations. For example, PCR-based methods, which are the simplest most frequently used methods, have limitations in detecting unknown mutations because the mutation sites vary. On the other hand, NGS-based methods do not have this limitation and have the highest resolution, but it is expensive and slow^13^. Aberrant DNA methylation is associated with the progression of various cancers, seeing as tumorigenesis and the phenotypes of cancer have been detected in patients’ blood, but detection rates and sensitivities were very low in the early stage^14^. Indeed, many analysis techniques and platforms should be developed and optimized for routine clinical diagnosis. Most of all, in addition to the requirement of a standard operating procedure (SOP) that many researchers agree with, any bias resulting from the small starting amount of cfDNA should be resolved to achieve efficiency, quality output and consistency^15^. Thus, it is proposed that if we use aberrant methylation of abundant circulating cfDNA fragments as cancer biomarkers, the sensitivity will be greatly increased by using only small amounts of plasma.

**Figure 1:**
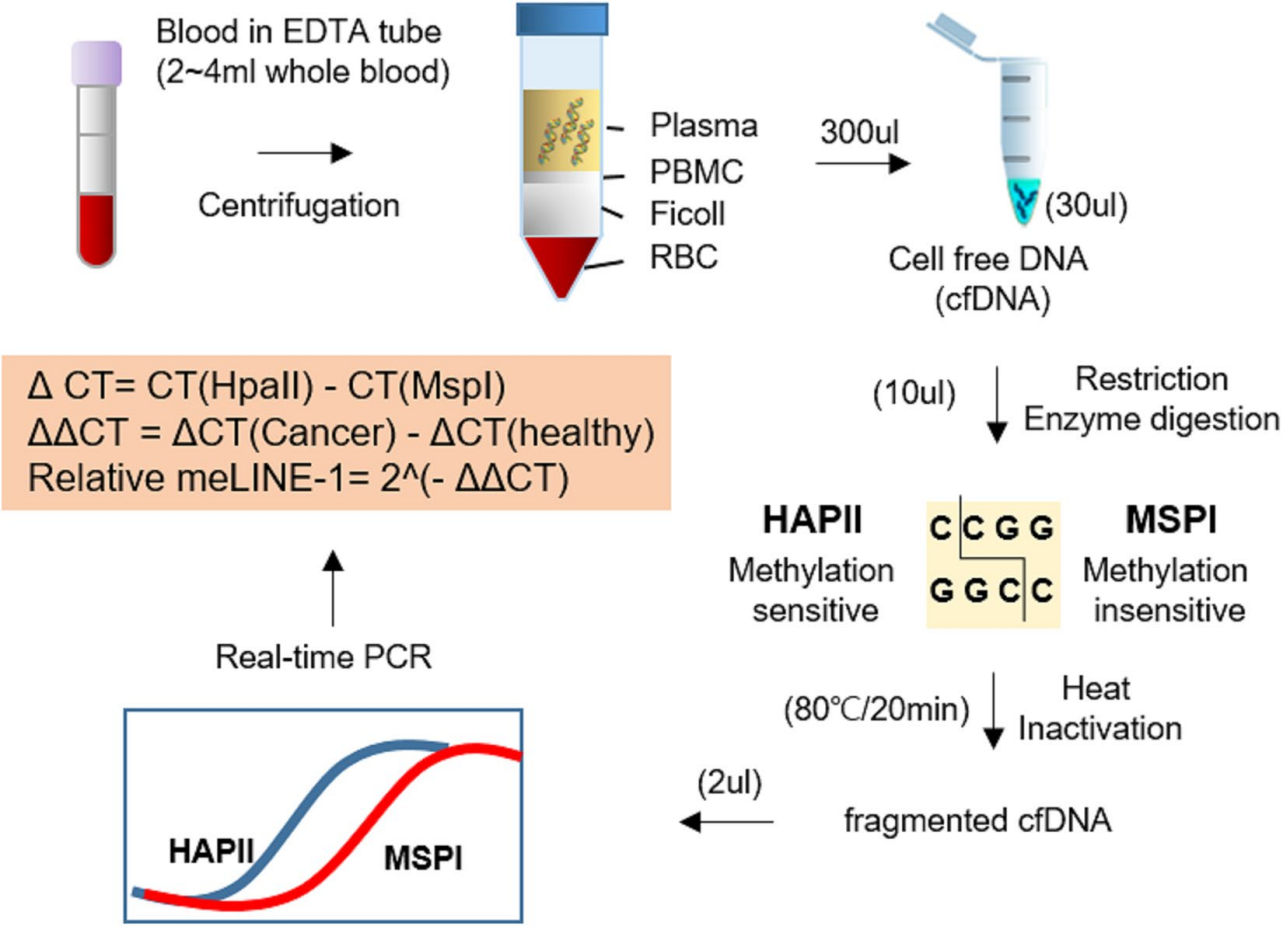
Experimental Scheme. The overall process is illustrated, from liquid biopsy through cell-free DNA isolation, and to the detection of LINE-1 methylation.

Long interspersed nuclear element-1 (LINE-1) constitutes ~17% of the human genome^16^. Since LINE-1 is a retrotransposon that is known to still be active, many studies have reported that LINE-1 expression and epigenetic regulation such as methylation status in cancer tissues is associated with several cancer characteristics, including types (i.e., colon, bladder, breast, and gastric cancer), progression, cancer risk, and poor prognosis^17–20^. In addition, the functional role of LINE-1 suggested by Hur *et al*. is that LINE-1 hypomethylation promotes liver metastasis by inducing expression of proto-oncogenes such as MET, RAB3IP, and CHRM 3 in colorectal cancer^21^. Thus, LINE-1 has been studied as a methylation marker associated with several types of cancer tissues. In this study, we aimed to determine for the first time whether canine cfDNA methylation can serve as an animal model system to screen for biomarkers of HBCs and whether LINE-1 methylation status can be used as a marker in liquid biopsies in both HBCs and CMTs (Fig. 1).

## Results

### Human and dog LINE-1

**Figure 2:**
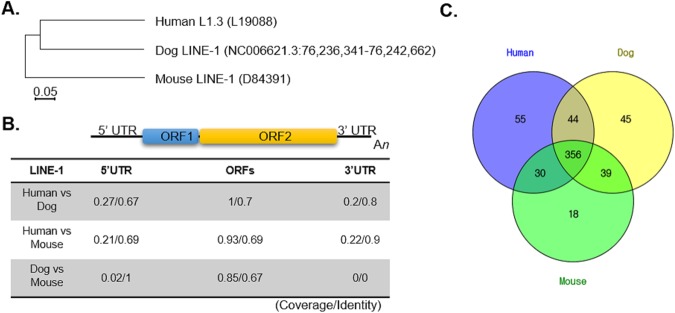
Comparison of LINE-1 sequences in the mouse, dog and human genomes. (**A**) Canine LINE-1 was more closely related with human LINE-1 than mouse LINE-1 in the comparison of the entire LINE-1 sequences. (**B**) A high similarity was found in ORF regions, whereas both 5′ and 3′UTRs showed low similarity. (**C**) In spite of low sequence similarity, many transcription factor-binding sites were conserved in the 5′UTR of the three LINE-1s.

Since approximately 20% of the human genome consists of LINEs (L1.3, GENBANK L19088), putatively active LINE-1 in the human genome and mINE-1 (D84391) in the mouse genome were chosen as reference sequences. The canine LINE-1 sequence was retrieved from the region of chromosome X: 76,236,341–76,242,662 [6,322 bp] by a BLAST search with human L1.3 in the dog genome (CanFam 3.1) (Fig. 2A). Two open reading frames (ORFs) of LINE-1 were well conserved among the three species’ LINE-1 sequences, while both 5′- and 3′-UTRs (untranslated regions) varied among the three organisms (Fig. 2B). Interestingly, in spite of lacking sequence similarity among the three 5′UTR regions of the LINE-1s, out of 589 predicted transcription factor binding sites (with higher than 85% similarity), 356 transcription factor binding sites were shared by all three LINE-1s, which means that they may be under similar regulation mechanisms (Fig. 2C). Overall similarity based on transcription factor binding sites in the regulatory regions of the three LINE-1s was higher between the human and dog, than between the human and mouse or the dog and mouse (Supplementary Data 1). Primers for both human and canine LINE-1 were designed based on the 5′UTR, where the internal promoter of LINE-1 presents. In silico PCR retrieved 84~85 bp size amplicons from 1305/2,686 loci (with/without “CCGG”) in human (hg38) and 78 bp amplicons from 94/108 loci in the dog reference genome (Canfam 3.1). As expected, there were many LINE-1 loci in both genomes with accumulated mutations that caused variations in the “CCGG” restriction enzyme cleavage sites.

### Differential methylation of the 5′UTR of LINE-1 between normal and mammary tumor cells

**Figure 3:**
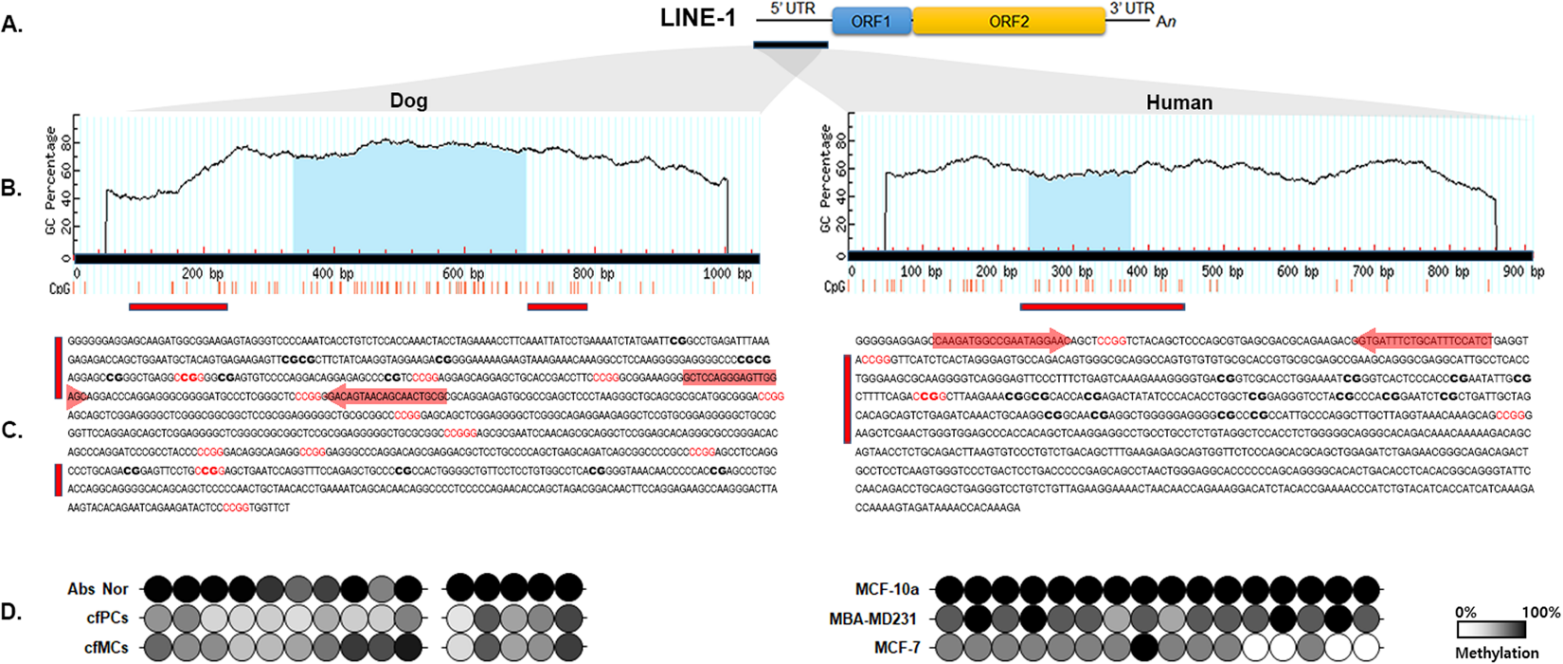
CpG island structure and methylation profile of LINE-1 5′UTR in breast cancer cell lines of human and dog. (**A**) LINE-1 structure (**B**) MethPrimer (http://www.urogene.org/cgi-bin/methprimer/methprimer.cgi) determined CpG Island structure in the 5′UTR of LINE-1 in both human and dog. (**C**) Regions for BSC-PCR (red-bar), primer binding sites for MSRED-rtPCR (red arrow) with restriction enzyme recognition sites (CCGG) are indicated. (**D**) BSC-PCR and sequencing revealed hypomethylation of LINE-1 in human and dog breast cancer cell lines. Methylation patterns of CpGs in the 5′UTR region of LINE-1 are shown in gray scale using lollipop diagrams.

We then found one CpG island (CGI) with a 136-bp size, GC > 50%, and an Obs/Exp ratio >0.8 from 910 bp in the 5′UTR of the human LINE-1 using MathPrimer (www.urogene.org) (Fig. 3A,B). Similarly, one CGI consisting of 341 bp in the flanking region was detected from the1,250 bp canine LINE-1 5′UTR. Primers for bisulfite conversion PCR (BSC-PCR) were targeted on the shore region of CGIs because the association of CGI shore methylation status with breast cancers, especially a highly aggressive basal-like B type, has been reported^22^.

BSC-PCR and sequencing revealed the methylation status of CGI regions consisting of 21 and 15 CpG sites, respectively, in both human and dog breast cancer-related cell lines (Fig. 3C). The ratios of C and T (methylated C and unmethylated C) on each CpG site were calculated and visualized by a lollipop diagram in gray scale. While all Cs were methylated in the 21 CpG sites of MCF 10a, human non-breast cancer cell lines, and normal dog mammary tissue, both human and dog breast cancer-related cell lines showed light gray or open circles, indicating hypomethylated cytosine bases (0~30%) (Fig. 3D). In particular, the human MCF7 HBC cell line showed significantly hypomethylated CpG sites. In addition, methylation levels on the fifteen CpG sites were surveyed by BSC-PCR in primary and metastatic CMT cell lines. Three of each primary and metastatic cell lines were pooled and subjected to BSC-PCR. As expected, the overall canine LINE-1 methylation was low in the CMT cell lines. Interestingly, canine LINE-1 methylation was lower in primary CMTs (~31.5%) than in metastatic CMTs (~43.9%).

### Methylation-sensitive restriction enzyme digestion (MSRED) followed by rtPCR

**Figure 4:**
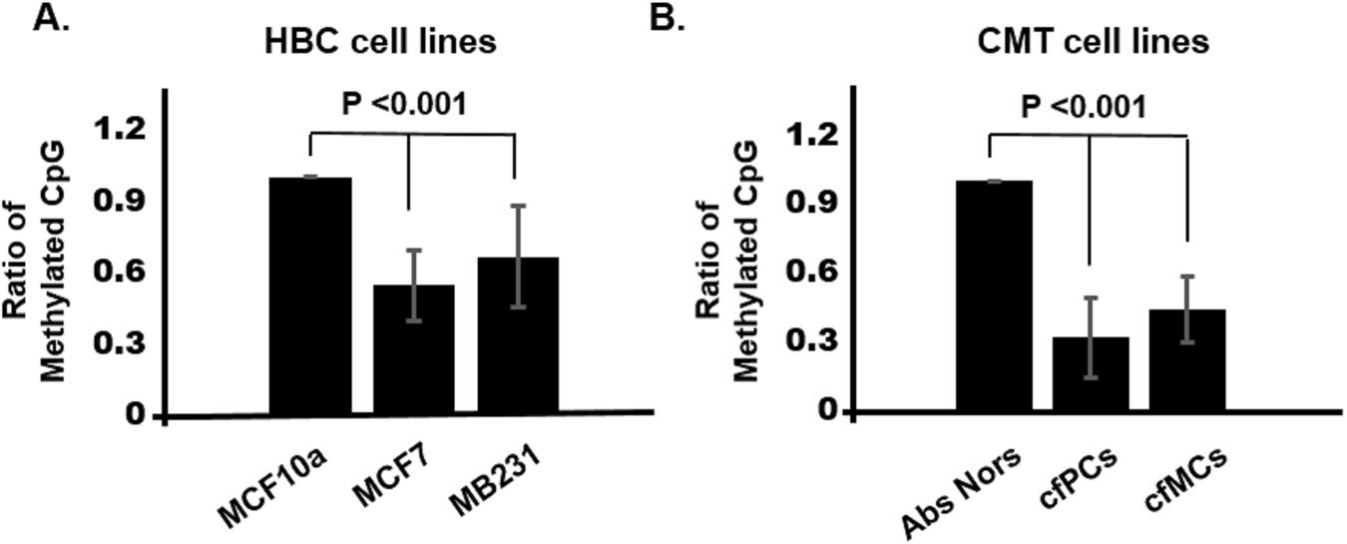
MSRED-rtPCR detected the hypomethylation of LINE-1 in breast cell lines of both human and dog. (**A**) Hypomethylated LINE-1 was confirmed with MSRED-rtPCR in the normal human breast cell line, MCF 10a and HBC related cell lines, MCF7 and MDA-MB-231. (**B**) The canine LINE-1 methylation level was analyzed in a primary mammary tissue and two sets of CMT cell lines (pooled with 3 primary- and 3 metastatic- tumors, respectively) and examined for methylation status by MSRED-rtPCR. LINE-1 hypomethylation levels were significantly lower in both human and canine tumor cell lines (p < 0.001).

We then employed the MSRED-rtPCR method to see whether the result from this method had a correlation with the one from the BSC-PCR. The LINE-1 hypomethylation determined by BSC-PCR sequencing from HBC and CMT cell lines was confirmed and visualized in a bar graph (Fig. 4). The difference in LINE-1 methylation found by BSC-PCR sequencing between the control and CMT cell lines tended to be amplified in the results of MSRED-rtPCR. The level of LINE-1 methylation in the MCF7 HBC cell line was significantly lower than in the MCF 10a normal cell line (p < 0.001). Moreover, the hypomethylation of LINE-1 was distinctly shown in the comparison between normal and cancer cell lines from dogs. The primary CMT cell lines showed the lowest level of methylation. In BSC-PCR, the methylation level discrepancy existing between normal and CMT cell lines was larger in the HBC cell lines than in the CMT cell lines, while it was the opposite in MSRED-rtPCR (Figs 3 and 4).

### Total amount of cfDNA was not significantly different in canine plasmas

**Figure 5:**
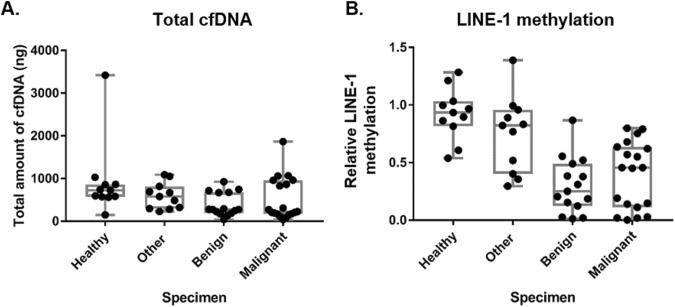
Significant LINE-1 hypomethylation was detected in the cfDNA of dog mammary tumors. (**A**) Total amounts of cell-free DNA (cfDNA) were similar among liquid biopsies of healthy controls, other cancers, and benign and malignant mammary tumors. cfDNA was isolated from 300 µl of plasma from healthy controls, and patients with other tumors, benign or malignant mammary tumors, and the total amount of cfDNA is visualized in box-and-whisker plots. (**B**) Box-and-whisker plots of relative methylation levels in cfDNA obtained from the plasma of healthy dogs, those with other various cancers, and mammary tumors (benign and malignant).

An increase in the amount of cfDNA in blood has been reported in various types of cancer patients. In this study, all blood specimens were processed for plasma and PBMC separation within 2 hr after drawing. cfDNA was successfully isolated from 300 µl plasma samples from 56 dogs. The yield of cfDNA was determined by a NanoDrop spectrophotometer. The absorbance ratio (A260/280) was used to assess the purity of the DNA in terms of protein contamination. Unexpectedly, the average amount of cfDNA isolated from healthy controls was 916.5 ng/ml, and the patient dogs with mammary tumors and other tumors showed a similar level (511.9 ng/ml). Thus, there was no significant difference in the total amount of cfDNA among the three groups (malignant, benign and healthy controls) of dogs, which might represent a discrepancy between humans and dogs (Fig. 5A).

### Measurement of LINE-1 methylation in clinical dog plasma specimens

Next, the procedure including cfDNA isolation, restriction enzyme digestion of cfDNA, and rtPCR was simplified by using equal volumes (e.g. equal volumes of plasma for cfDNA isolation, equal volumes of cfDNA for restriction enzyme digestion, and equal volumes of fragmented cfDNA for rtPCR) (Fig. 1). This procedure is helpful to reduce bias and errors that might be generated during analytical steps. In addition, detection of LINE-1 methylation can compensate for another risk caused by a low amount of cfDNA.

**Figure 6:**
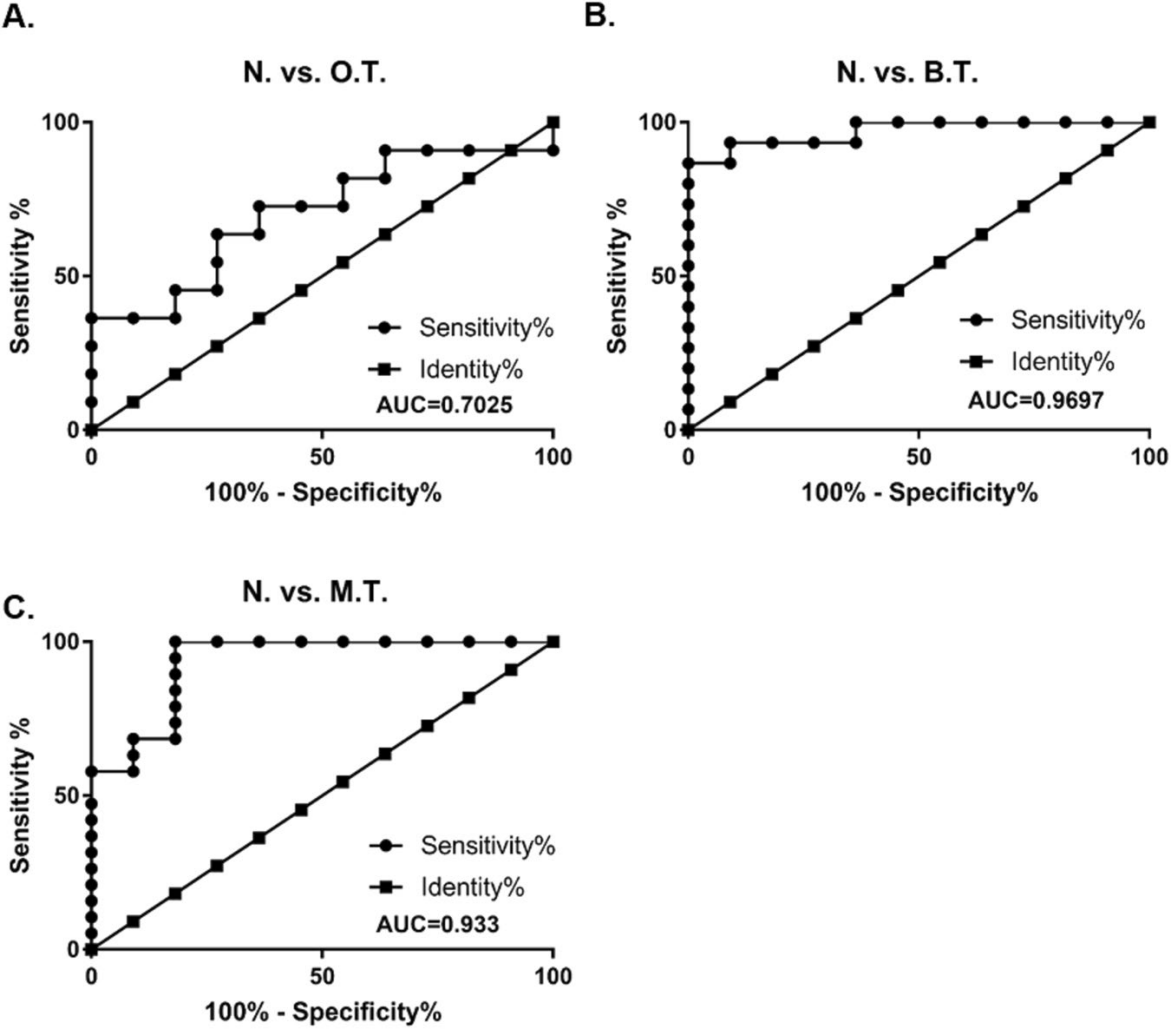
Receiver operating characteristic (ROC) curve for the comparison of healthy controls with (**A**) other dog tumor types (N vs. O.T.), (**B**) benign mammary tumor (N vs. B.T.) and (**C**) malignant mammary tumor (N. vs M.T.). The area under the curve was calculated.

A total of 56 dog specimens, from healthy controls and dogs with benign tumors, mammary cancers and other types of cancers, are summarized in Table 1. Theoretically, no amplification is expected from MspI digestion followed by rtPCR because all target sequences should be cut in the middle by MspI, meaning that the DNA cannot be amplified. However, there was still amplicons, possibly due to the existence of LINE-1 with mutations in the CCGG region. The relative ratio of methylation was calculated by a modified delta-delta CT method (2^−[delta][delta]CT^). The delta CT = CT(HpaII)-CT(MspI) and delta-delta CT = deltaCT(Cancer) − deltaCT(healthy). While the related methylation level in healthy controls is ~1, in the malignant CMT, it is lower than 0.4 (Fig. 5B). Unexpectedly, the lowest methylation level was detected in benign tumors (~0.2). Hypomethylated LINE-1 was also detected in other types of cancer samples but at insignificant levels. This significant discrepancy (p < 0.001) in LINE-1 methylation between healthy controls and mammary tumors generated an ROC curve with an area under the curve (AUC) higher than 0.9 (Fig. 6).

**Table 1:** Summary of dog blood specimen.

Group	F/FS(M/NM)	Age	Breed
Healthy	6/5	0.3–12	Maltese, Schnauzer, Coker Spaniel, Poodle, Shih-tzu
Benign	10/5	5–15	Shih-Tzu, Schnauzer, Poodle, Maltese, Coker Spaniel, Dachshund, Spitz
Other C.	2/4 (3/2)	6–14	Pekingnese, Maltese, Shih-tzu, Schnauzer, Yorkshire Terrier, Coker Spaniel, Mix
Malignant	5/14	6–15	Great Pyrenees, Schnauzer, Maltese, Dachshund, Yorkshire Terrier, Chihuahua, jindo, Miniature Pinscher, Coker Spaniel, Poodle, Welsh Corgi, Shih-Tzu

F: female.

FS: female spay.

M: male.

NM: Neutering male.

### Comparative analysis for HBCs diagnosis

**Figure 7:**
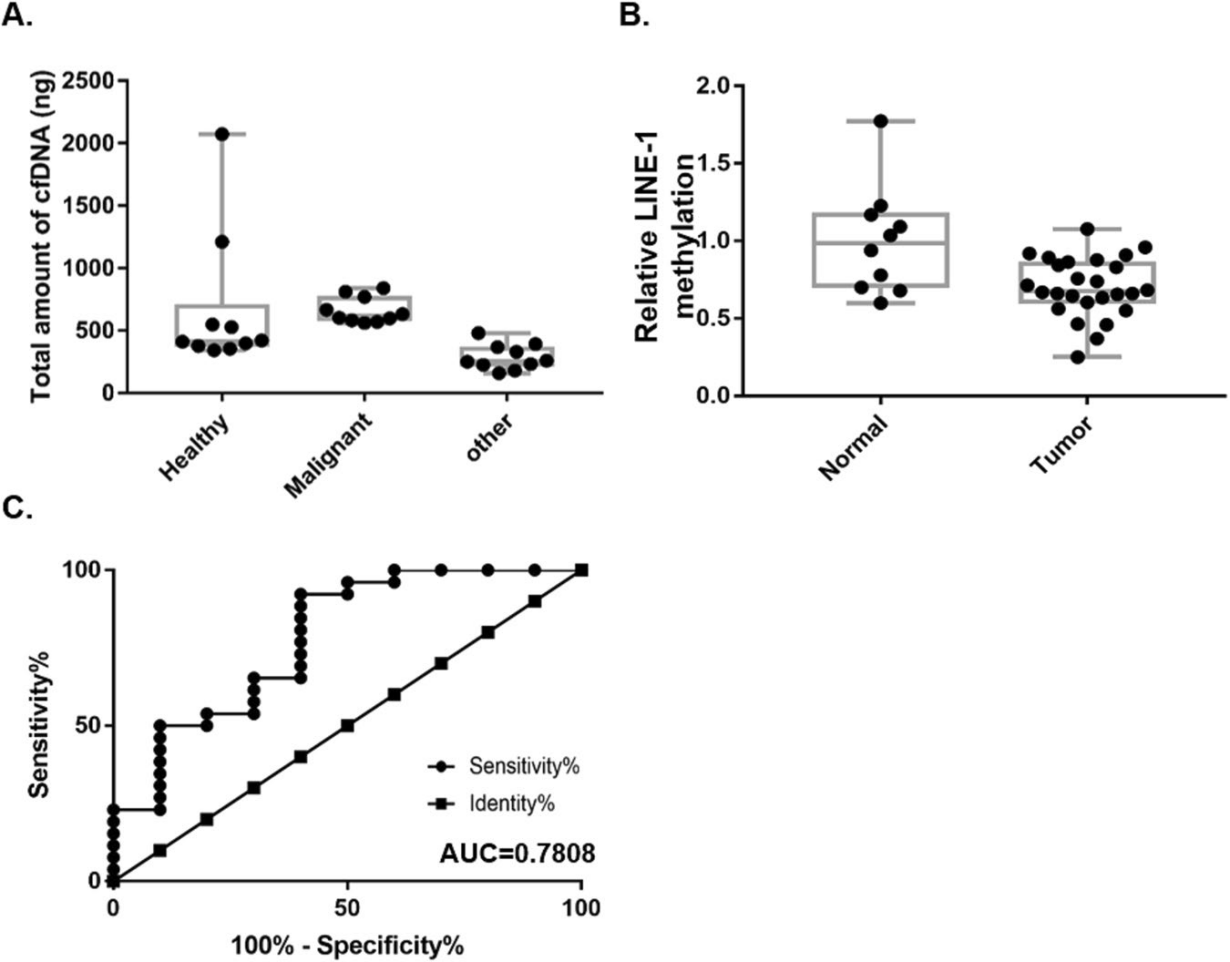
Implementation of the comparative strategy in human breast cancer diagnosis. (**A**) Total amount of cfDNA isolation. (**B**) Human LINE-1 methylation in cfDNA obtained from healthy control and breast cancer patients’ plasma. (**C**) ROC curve for human LINE-1 methylation in breast cancer diagnosis.

We further tested 36 human plasma specimens obtained from ten healthy controls and from 26 patients with breast cancers of various stages, types and ages (Table 2). The total amount of cfDNA isolated from 300 µl of plasma was significantly higher from HBC patients than from healthy controls. This result is consistent with previous studies (Fig. 7A). The difference in LINE-1 methylation levels between healthy controls and HBCs was smaller in humans than in dogs but the methylation levels in the healthy controls were still significantly lower than for HBC patients (Fig. 7B). LINE-1 methylation was ~0.7 in HBC samples, while for healthy controls, it was ~1 (Fig. 7B). ROC curves were constructed, and the AUC was determined to assess the diagnostic values of LINE-1 methylation. As shown in Fig. 7C, ROC analyses revealed that the AUC of LINE-1 methylation was 0.7808 (0.5996–0.962), with a p value of 0.01.

**Table 2:** Human blood specimen.

ID	Group	Age	Sex	Stage	ER	PR	HER-2
BB003P	Healthy	41	F				
BB004P	Healthy	44	F				
BB005P	Healthy	21	F				
BB006P	Healthy	38	F				
BB007P	Healthy	38	F				
BB008P	Healthy	31	F				
BB009P	Healthy	26	F				
BB011P	Healthy	42	F				
BB012P	Healthy	48	F				
BB015P	Healthy	45	F				
BC009P	Breast cancer	32	F	0	+	−	−
BC018P	Breast cancer	55	F	0	−	−	+
BC022P	Breast cancer	54	F	0	+	+	−
BC001P	Breast cancer	57	F	IA	+	−	+
BC004P	Breast cancer	46	F	IA	+	+	−
BC016P	Breast cancer	47	F	IA	−	−	−
BC023P	Breast cancer	48	F	IA	+	+	−
BC035P	Breast cancer	34	F	IA	−	−	−
BC038P	Breast cancer	41	F	IA	+	+	−
BC041P	Breast cancer	45	F	IA	+	+	−
BC042P	Breast cancer	41	F	IA	+	+	−
BC043P	Breast cancer	47	F	IA	+	+	−
BC055P	Breast cancer	43	F	IA	+	+	−
BC058P	Breast cancer	54	F	IA	−	−	−
BC013P	Breast cancer	40	F	IB	−	−	−
BC005P	Breast cancer	35	F	IIA	+	+	−
BC019P	Breast cancer	36	F	IIA	+	+	−
BC024P	Breast cancer	45	F	IIA	+	+	−
BC026P	Breast cancer	64	F	IIA	+	+	+
BC034P	Breast cancer	44	F	IIA	+	+	+
BC056P	Breast cancer	51	F	IIA	+	+	−
BC003P	Breast cancer	38	F	IIB	+	+	+
BC007P	Breast cancer	45	F	IIB	+	+	−
BC032P	Breast cancer	44	F	IIB	−	−	−
BC060P	Breast cancer	42	F	IIB	−	−	−
BC031P	Breast cancer	38	F	IIIC	+	+	−

Age, histological stages, and three major molecular markers (ER, PR, HER-2) are indicated.

## Discussion

Currently, the imaging modalities of mammography and ultrasound are routinely used for breast cancer diagnosis. With recent development gene profiles such as PAM50 have been approved by the FDA for the prognosis of breast cancers^23^. However, the need for early diagnosis with high sensitivity and specificity has led to a search for biomarkers based on simple liquid biopsies using blood or urine. A number of methylation biomarkers that can be used to distinguish between healthy controls and breast cancers using cfDNA have recently been highlighted^8,24^. Most studies have targeted the promoter methylation of specific genes (e.g., RASSF1A, APC, and SOX17) for which the expression has been previously defined in cancers^25^. Although cfDNA has great potential to be used as a biomarker for breast cancer detection, some hurdles such as bias and inconsistency in the results have been caused by the use of small amounts of cfDNA. Varying methylation profiles found in certain gene promoters need to be overcome before cfDNA can be used in HBC diagnosis. On the other hand, instead of studying individual genes and their epigenetic aberrations, there have also been attempts to search for a candidate biomarker using cfDNA and repetitive elements, such as short interspersed nuclear element (SINE, represented by ALU)^26^. SINEs that are small and non-autonomous transposable element has been suggested as prognostic markers of canine mammary tumor without association of DNA methylation^27,28^, whereas there have been no previous attempts to use LINE-1s and its methylation as a biomarker for the liquid biopsy of breast cancer.

In this study, we first compared three LINE-1 sequences in human, mouse and dog. Two ORF regions(ORF1 and ORF2) are well conserved, but both the 5′ and 3′ UTRs differ in all three organisms. Although the 5′UTRs where the regulatory sequences of LINE-1 are presented vary among the organisms, 356 transcription factor binding sites were shared by all three sequences. In this LINE-1 sequence comparison, we could confirm the existence of a higher system similarity between human and dog than between human and mouse. Moreover, CGI regions with 136-bp and 341-bp sizes were hypomethylated in both CMT and HBCs. Thus, we tried to develop the methylation level of canine LINE-1 as a biomarker of CMT and expanded it to HBCs, although the sample size was small since this was a pilot study. We first verified the suitability of the MSRED-rtPCR method for methylation detection. There was a small difference in the level of methylation between MSRED-rtPCR and BSC-PCR, but the MSRED-rtPCR method successfully represented the methylation levels and tended to amplify the difference between healthy controls and cancers (Figs 3 and 4).

The use of LINE-1 as a target sequence is a double-edged sword for a candidate biomarker using cfDNA. High copy numbers of LINE-1 in the genome will be of benefit when the liquid biopsy sample amount is small. However, high similarity of LINE-1 elements with sequence mutations might add complexity to the interpretation of the data. Although an increase in total cfDNA in various human cancers has been reported^29^, unexpectedly, the total amount of cfDNA isolated from equal volumes of plasma (with/without CMT) was not significantly different between canine healthy controls and CMT patients (Fig. 5A). This might be different between dogs and humans or between CMT and HBC. The amount of canine cfDNA has been tested and reported in many studies, where the level of cfDNA was varied depending on isolation procedure, particularly storage hours at room temperature after collection. Deborah Burnett *et al*. tested a few variables such as disease condition, and storage temperature and hours after collection and reported that 550–610 ng/ml cfDNA was isolated from normal and 622–818 ng/ml from diseased animals^30^. It isn’t much different from our result when one outlier whose amount showed more than 3 µg in the healthy group is eliminated. The outlier showing more than 3 µg of cfDNA is speculated to have had genomic DNA contamination from blood cells ruptured during collection or plasma separation (Fig. 5A).

Although the amount of cfDNA can be diverse depending on the isolation method, our data clearly showed that the methylation levels of canine LINE-1 in CMTs were significantly lower than in healthy controls and could be detected in small volumes of blood (Fig. 5B). In addition, hypomethylation was more dramatically shown in CMT than HBC (Figs 6 & 7). This can be explained by discrepancies existing at any level, such as between human and dog, CMT and HBC, or human LINE-1 and canine LINE-1. Since there was no information available regarding cancer stages for the CMT specimens, we could not compare CMT and HBC in similar stages directly. However, we could also find no correlation among HBC stages and human LINE-1 methylation levels (Table 2). Another explanation might be the differences between the numbers of target LINE-1 sequences on the dog and human genomes and mutation rates. The putative targets include only 203 loci in the dog genome, and half of the sequences retained the CCGG site. However, 3,991 loci are found in the human genome but only one-third of the loci retain the CCGG sites, resulting in low sensitivity. Since the final goal of this study is to develop biomarkers for the liquid biopsy of both HBC and CMT, there is still a lot of room for technical improvement. A biomarker for clinical use should be supported by good sensitivity and specificity. To this end, target sequence optimization within LINE-1 might be a good course of action to improve both specificity and sensitivity. We have tested a few loci on the 5′UTR of LINE-1 to detect methylation status and retrieved results varying depending on loci. On the other hand, the joint or separate use of different types of repetitive elements families, such as endogenous retrovirus of which expressions have known to be associated with HBC might help to raise the AUC up in HBC^31^.

Interestingly, the methylation levels of LINE-1 were even lower in benign tumors (Fig. 5B). Thus, the comparison of healthy and benign tumors showed better diagnostic power (AUC 0.97, Fig. 6B) than the comparison of healthy and malignant tumors (AUC 0.93, Fig. 6C)). It is important to understand the difference between benign and malignant tumors. Hartmann *et al*. (2005) has already reported an association between benign breast disease and malignant breast cancer, and some proteins have been studied as risk factors for malignancy^32^. Moreover, it has been reported that a high level of expression of molecular biomarkers such as estrogen receptors (ERs) and progesterone receptors (PRs) in benign tumors are associated with an increased risk of breast cancers^33^. That is, there might be transition from benign breast tumors to malignant breast cancer at least in some cases^34^. Thus, a lower methylation status of LINE-1 in benign CMT could represent early changes in LINE-1 DNA methylation.

Besides the methylation level of LINE-1 in cfDNA as a diagnostic biomarker for breast cancer, its origin, release mechanism, and biological significance should be investigated. Multiple structures such as exosomes and nucleosomes, or biological mechanism such as necrosis and apoptosis have been known as the source of cfDNA^35^. Indeed, further investigation is guaranteed in the properties of LINE-1, such as methylation, stability, and integrity of plasma cfDNA within HBC or CMT.

## Conclusions

A comparative approach using naturally occurring CMTs is a suitable model for the study of HBC biomarkers. We tested the use of repetitive, abundant, yet highly cancer-related LINE-1 methylation in the cfDNA isolated from small amounts of plasma from patients with CMT and HBC. Canine LINE-1 hypomethylation clearly differentiated CMTs from healthy controls, and the same approach also worked for HBCs. Further development with a larger sample size, different stages and different types of HBCs is expected to provide a useful procedure regarding restriction enzyme cleavage sites and primer binding sites for LINE-1 methylation to be used as a feasible biomarker for both CMT and HBC.

## Methods

### Specimen

This study was reviewed and approved by the Seoul National University Institutional Review Board/Institutional Animal Care and Use Committee (IACUC# SNU-170602-1/IRB#SNU 16-10-063) and all methods were performed in accordance with the relevant guidelines and regulations.

#### Dogs

Companion dogs diagnosed with mammary tumors (benign and malignant), other types of tumors, and healthy controls without symptoms based on the results of blood chemistry were enrolled in this study. A total of 56 dogs, summarized in Table 1, consisting of various ages and breeds as well as cancer categories were enrolled. Two to four ml of blood was collected in EDTA anti-coagulant tubes (Vacuette®, Greiner Bio-One, Kremsmunster, Austria) depending on the size of each dog.

#### Humans

Twenty-six breast cancer patients and 10 healthy controls were enrolled in this study from Samsung Medical Center in Seoul. The IRB was approved by both the Samsung Medical Center (# SMC2016-07-129) and Seoul National University (IRB#SNU 16-10-063). After written informed consent, blood samples were collected from breast cancer patients who were diagnosed with primary breast cancer and were scheduled to undergo surgery. Healthy control individuals eligible for inclusion had no abnormal findings on breast examination. From each breast cancer patient and control individual, fresh whole blood (4~6 ml) was collected by standardized venipuncture. Participant information is listed in Table 2.

### Plasma separation and cell-free DNA isolation

2 hours after blood collection, plasma and peripheral blood mononuclear cells (PBMCs) were separated using a standard, previously validated protocol. Briefly, blood was applied to an equal volume of Ficoll-Paque™ PLUS (GE Healthcare, Orsay, France) and centrifuged for 30 min, at 500 g, 18 °C without a brake. Plasma was collected from the supernatant and PBMCs were obtained from the central white band of the gradient. Plasma was stored at −80 °C until cfDNA isolation.

### Transcription factor analysis

The regulatory region (2,000 bp of 5′UTR) of three LINE sequences (L19088, D84391 and CanFam 3.1, ChrX: 76,236,341-76,238,340) were submitted to the PROMO website (http://alggen.lsi.upc.es/cgi-bin/promo_v3/promo/promoinit.cgi?dirDB=TF_8.3) and transcription factor binding sites were searched for with default options (85% similarity). The data consisting of name, matrix, loci and similarity was retrieved and the numbers of each transcription factor binding sites were counted based on matrix. Scatter plots and correlation coefficients were analyzed using Excel function

### Cell culture

MCF7 and MDA-MB-231 were maintained in Dulbecco’s modified Eagle’s medium (DMEM) (HyClone, GE Healthcare Life Sciences, USA), containing 10% fetal bovine serum (FBS) (JCBIO Co. LTD, Korea) and antimycotics/antibiotics (Gibco, USA), while MCF 10A was maintained in DMEM/F12 (HyClone), containing 10% FBS and antimycotics/antibiotics. Six CMT cell lines (CHMp, CHMm, CTBp, CTBm, CIPp and CIPm) were purchased from Nobuo Sasaki Lab., where the cell lines were originally established^36^, and maintained in RPMI‐1640 with 10% FBS and gentamycin (Sigma, USA).

### DNA isolation

Genomic DNA was isolated from three human breast cancer-related cell lines, six canine mammary gland tumor cell lines and a normal dog mammary gland tissue using the DNeasy blood & tissue kit (Qiagen) according to the manufacturer’s guideline. Genomic DNA was eluted into 50 µl buffer EB. The cfDNA was isolated from 300 µl of plasma using the Qiagen cfDNA isolation kit (Qiagen) according to the manufacturer’s manual, and finally eluted into 30 μl AVE buffer. All cfDNA and genomic DNA samples were quantified on a Nanodrop spectrophotometer and stored at −80 °C until use.

### Bisulfite conversion PCR and sequencing

Each aliquot of 500 ng genomic DNA was subjected to bisulfite conversion using an EZ DNA Methylation-Lightning Kit according to the manufacturer’s protocol (Zymo Research Corporation, USA). The methylation status of the 5′UTR region of LINE-1s was determined by the bisulfite conversion-polymerase chain reaction (BS-PCR). The following primers were used for the PCR: dog LINE-1 (78 bp), forward primer: 5′-GCGCGCAGTTGCTGTTACTGT; reverse primer: 5′-GCTCCAGGGAGTTGGAGCAG and human LINE-1 85 bp), forward primer: 5′- CAAGATGGCCGAATAGGAAC; reverse primer: 5′- CAGATGGAAATGCAGAAATCAC. Amplicons were extracted from agarose gels using a QIAquick Gel Extraction Kit (Qiamap). Sequencing service was provided by Solgent Co. Ltd. (Solgent Co. Ltd., Seoul, Korea).

### Methylation-sensitive restriction enzyme digestion (MSRED) and rtPCR

To minimize the variations caused by low amounts of cfDNA, we reduced the DNA recovery steps from cfDNA isolation, through the detection of LINE-1 methylation levels. Equal volumes of cfDNA were subjected to MSRED using MspI and HpaII (Thermo Fisher Scientific, USA). Genomic DNA (500 ng) and cfDNA (10 µl) were digested by 5 units of the restriction enzymes HapI and MspI, respectively, with buffer Tango in a 20-µl reaction mixture at 37 °C for 3 hours, followed by 80 °C heat inactivation for 20 minutes. Two µl of the DNA digestion mixture was used as the template for rtPCR. rtPCR was performed using the following conditions: 95 °C for 3 min and then 40 cycles of 95 °C for 20 s, 58 °C for 20 s, and 72 °C for 30 s, with a final extension at 72 °C for 5 min in a Bio-Rad Real-Time PCR detection System (Bio-Rad, USA). Each sample was duplicated, and each run included a no-template control. The scheme of the procedure is illustrated in Fig. 1.

### Calculations and Statistics

The relative amount of methylated LINE-1 was calculated by a modified 2^−[delta][delta]CT^ method: Delta CT = CT(HpaII) − CT(MspI), and delta-delta CT = deltaCT(Cancer) − deltaCT(healthy). The significance of the methylated LINE-1 level in the plasma was determined by a one-way ANOVA test. The ROC value, sensitivity and specificity were calculated by GraphPad PRISM 7 software (GraphPad Software, La Jolla, CA).

## Electronic supplementary material

Supplementary data 1

## Data Availability

All data generated or analyzed during this study are included in this published article and its Supplementary Information Files.
