# Author Correction: Methylation of LINE-1 in cell-free DNA serves as a liquid biopsy biomarker for human breast cancers and dog mammary tumors

**DOI:** 10.1038/s41598-019-53895-8

**Published:** 2019-11-20

**Authors:** Kang-Hoon Lee, Tae-Jin Shin, Wan-Hee Kim, Je-Yoel Cho

**Affiliations:** 1 Department of Biochemistry, BK21 Plus and Research Institute for Veterinary Science, School of Veterinary Medicine, Seoul National University, Seoul, South Korea; 2 Department of Veterinary Clinical Sciences, College of Veterinary Medicine and Research Institute for Veterinary Science, Seoul National University, Seoul, Republic of Korea

Correction to: *Scientific Reports* 10.1038/s41598-018-36470-5, published online 17 January 2019

Soo-Youn Lee was incorrectly listed as an author in the original version of this Article and has now been removed from the author list. In addition, the Acknowledgements section in the original version of this Article was incomplete and now reads:

“This research was supported by the Bio & Medical Technology Development Program of the National Research Foundation (NRF) funded by the Ministry of Science and ICT (#2016M3A9B6026771) and partially by (#2016R1A6A3A11932951). The authors wish to thank Dr. Sun-Young Hwang at the Haemaru referral animal hospital for assistance in providing some of the clinical specimens.

The authors also wish to thank Isaac Kim, Jai Min Ryu, Hee Jun Choi, Jae-Myung Kim, Se Kyung Lee, Jonghan Yu, Seok Won Kim, Seok Jin Nam, Jeong Eon Lee and Soo-Youn Lee at the Samsung Medical Center for assistance in providing the valuable clinical specimens.”

These errors have now been corrected in the HTML and PDF versions of the Article, and in the accompanying supplementary material.

