# Supplementary material for: Methylation of LINE-1 in cell-free DNA serves as a liquid biopsy biomarker for human breast cancers and dog mammary tumors
Source: Sci Rep. 2019 Jan 17;9:175. doi: 10.1038/s41598-018-36470-5 (PMC6336845; doi:10.1038/s41598-018-36470-5)
Supplement: Supplementary file 1 — Supplementary data 1 [file 41598_2018_36470_MOESM1_ESM.pdf]

Supplementary data 1 for

Methylation of LINE-1 in cell-free DNA serves as a liquid biopsy biomarker for human breast cancers and dog mammary tumors

Kang-Hoon Lee[1], Tae-Jin Shin[1], Wan-Hee Kim[2], and Je-Yoel Cho[1]*

[1]Department of Biochemistry, BK21 Plus and Research Institute for Veterinary Science, School of Veterinary Medicine, Seoul National University, Seoul, South Korea

[2]Department of Veterinary Clinical Sciences, College of Veterinary Medicine and Research Institute for Veterinary Science, Seoul National University, Seoul, Republic of Korea

Address correspondence to:

*Je-Yoel Cho, DVM, PhD

Professor,

Department of Biochemistry

College of Veterinary Medicine, Seoul National University

1 Gwanak-ro, Gwanak-gu, Seoul, Korea 151-742

Tel.: +82-02-880-1268, Fax: +82-2-886-1268

E-mail: jeycho@snu.ac.kr

Supplement data 1. Transcription factor binding sites analysis

A) Correlation of TFBS profiles in LINE-1 among three different species

Correlation coefficient		
Hu/Do	Hu/Mo	Do/Mo
0.8503757	0.8380058	0.7270415

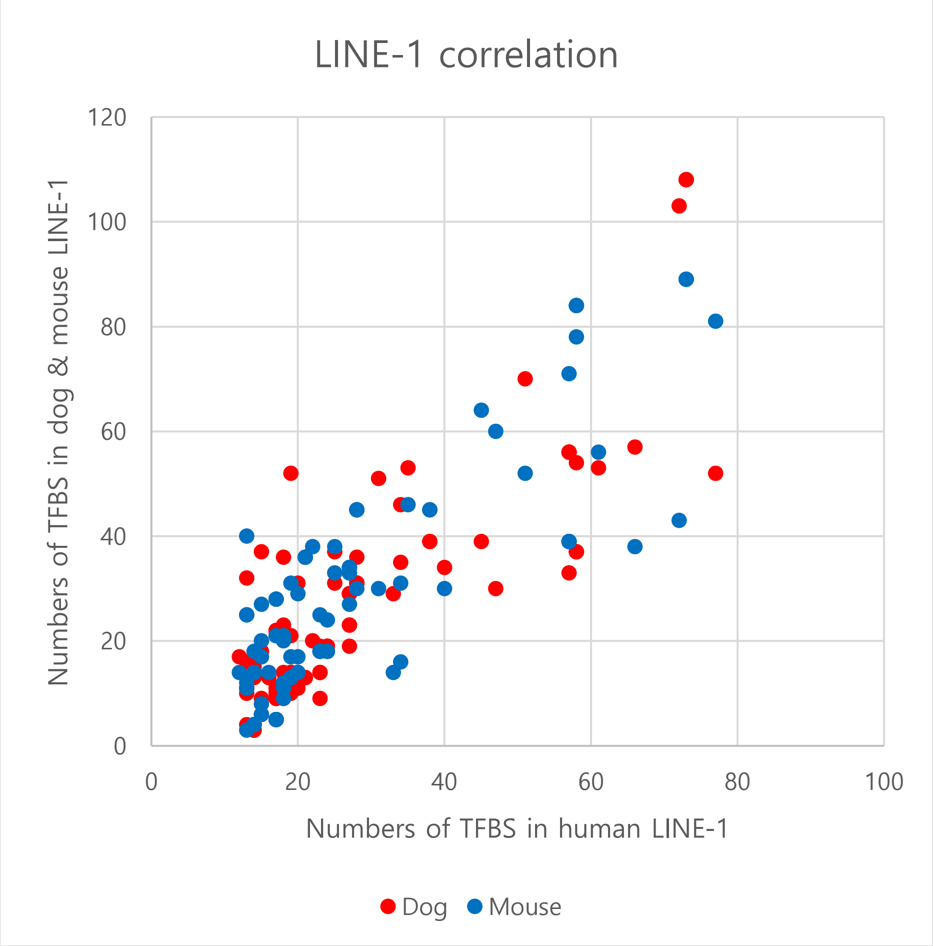

B) List of top 100 frequent TFBSs in LINE1s

TFBS[Matrix]	Human	Dog	Mouse
MF3 [T00507]	77	52	81
FACB [T02841]	73	108	89
Elk-1 [T00250]	73	108	89
p300 [T01427]	73	108	89
HNF-3 [T02277]	72	103	43
C/EBPalpha [T00107]	66	57	38
MYB2 [T02536]	61	53	56
Zic3 [T04671]	58	37	84
Zic1 [T04669]	58	37	84
Zic2 [T04670]	58	37	84
HOXA3 [T00378]	58	54	78
BR-C Z2 [T01478]	57	56	39

C/EBPdelta [T00109]	57	56	39
unc-86 [T01882]	57	56	39
POU1F1a [T00691]	57	56	39
Msx-1 [T02072]	57	33	71
R2 [T00712]	51	70	52
RC2 [T00724]	47	30	60
Pax-6 [T00682]	45	39	64
Cutl1 [T02042]	40	34	30
Ncx [T04368]	38	39	45
Cdx-1 [T01484]	38	39	45
ZF5 [T02349]	35	53	46
DEF:GLO:SQUA [T03217]	34	35	16
GA-BF [T00297]	34	46	31
C/EBP [T01386]	33	29	14
EIIaE-A [T00246]	31	51	30
CREMtau [T01309]	28	31	45
CREMtau2 [T02109]	28	31	45
myogenin [T00528]	28	31	45
HELIOS [T06012]	28	36	30
CREMtau1 [T02108]	28	31	45
NF-1 [T01298]	27	23	33
c-Ets-1 [T00112]	27	29	34
STAT5A [T04683]	27	29	34
STAT4 [T01577]	27	29	34
HMG I(Y) [T02368]	27	29	34
E47 [T00207]	27	23	33
CP2 [T00152]	27	19	27
Nkx2-1 [T00857]	25	37	33
Nrf2:MafK [T05666]	25	31	38
AGL3 [T03025]	24	19	18
POU3F2 [T00630]	24	19	18
AP-2alphaA [T00035]	24	18	24
DSXM [T00956]	23	14	18
DSXF [T00955]	23	14	18
Pax-4a [T02983]	23	19	18
WT1 I [T01840]	23	9	25
MyoD [T00526]	22	20	38
Pax-9b [T03594]	21	13	36
ABI4 [T05743]	21	13	36
Pax-9a [T03593]	21	13	36
NF-X3 [T01514]	20	14	29
TRM1 [T05311]	20	31	14
LF-A1 [T00467]	20	11	17
TGGCA-binding protein [T00832]	19	14	13
NF-1 [T00538]	19	14	13
NHP-1 [T00621]	19	10	17
LIM1 [T04817]	19	14	13
MYBAS1 [T05553]	19	52	13

DBP [T00183]	19	21	31
VDR [T00885]	18	9	21
COE1 [T01112]	18	36	20
NFI/CTF [T00094]	18	14	12
ABF1 [T00056]	18	10	11
c-Myb [T00137]	18	11	9
DEF:GLO [T03216]	18	23	12
PR A [T01661]	17	22	28
C/EBPalpha [T00108]	17	9	5
Alfin1 [T04733]	17	11	21
C/EBPalpha [T00104]	17	10	5
USF-1 [T00875]	17	11	21
HNF-3beta [T02344]	17	22	28
C/EBPbeta [T00017]	17	10	5
C/EBPalpha [T00105]	17	9	5
PR B [T00696]	17	22	28
C/EBPbeta [T00581]	17	10	5
C/EBPbeta [T00459]	17	9	5
POU3F1 [T00969]	16	13	14
GR-alpha [T00337]	15	17	27
Spz1 [T04668]	15	9	20
Dl [T00196]	15	18	8
LCR-F1 [T01599]	15	6	6
p53 [T00671]	15	37	17
TCF-4E [T02878]	14	3	4
TFIIB [T00818]	14	13	14
YY1 [T00278]	14	4	14
LEF-1 [T02905]	14	3	4
Pax-2a [T00678]	14	15	18
TCF-1A [T00999]	14	3	4
YY1 [T00915]	14	4	14
YY1 [T00865]	14	4	14
HNF-3beta [T02513]	13	16	12
Nkx2-1 [T00856]	13	10	13
POU2F2 (Oct-2.1) [T00646]	13	13	11
MATalpha2 [T00487]	13	4	3
ENKTF-1 [T00255]	13	11	25
f(alpha)-f(epsilon) [T00287]	13	32	40
NF-1 [T00537]	13	11	25
COE2 [T05006]	12	17	14
